# Cambaloid millipedes of Tasmania, Australia, with remarks on family-level classification and descriptions of two new genera and four new species (Diplopoda, Spirostreptida)

**DOI:** 10.3897/zookeys.827.32969

**Published:** 2019-03-05

**Authors:** Robert Mesibov

**Affiliations:** 1 West Ulverstone, Tasmania 7315, Australia Unaffiliated West Ulverstone Australia

**Keywords:** Diplopoda, Spirostreptida, Cambalidae, Iulomorphidae, Tasmania, Australia

## Abstract

The Southern Hemisphere cambaloid millipede genera are here assigned or re-assigned to the families Cambalidae Bollman, 1893 and Iulomorphidae Verhoeff, 1924. *Tasmanocambala* is erected for the three Tasmanian cambalids, *T.greeni***gen. n., sp. n.** (type species), *T.tasmanica***sp. n.** and *T.taylori***sp. n.** The new genus is distinguished by a thin, transverse tab at the tip of the anterior gonopod telopodite with a comb of setae immediately behind the tab. The iulomorphid *Talomiusweldensis***gen. n., sp. n.** is described from a single site in Tasmania’s southern mountain district. The new species is unusual among the Australian Iulomorphidae in having a fully-developed, ambulatory leg 1 in the male, and small, compact gonopods.

## Introduction

The classification of the cambaloid spirostreptidans has long been unsettled:

“*Suborder Cambalidea. The systematics of this group are, and will for a long time remain, in a state of particular confusion...*” (Hoffman 1980: 82).

“*Throughout this century great confusion has reigned regarding the classification of the ‘Cambalida’...*” (Jeekel 1985: 101)

“*Having had very little experience with cambalidans, I am not in a position to resolve this chaos...*” (Shelley 2003: 191).

“*Confusion still reigns in the various classification databases available on the WWW, and the position of all the “cambalidan” families are chaotic...*” (Korsós and Johns 2009: 2).

Especially confusing has been the systematics of the Australasian cambaloid genera, which have even been placed in different orders (Mauriès 1987). In his classification of the Diplopoda, Hoffman (1980) placed three of the Australasian cambaloid genera in Cambalidae Bollman, 1893 and the rest in Iulomorphidae Verhoeff, 1924, which he described as “an assemblage of incongruities” (Hoffman 1980: 84). The 10 Southern Hemisphere iulomorphid genera were “provisionally referred here pending revisionary studies” (Hoffman 1982: 699).

Jeekel (1985) accepted Hoffman’s grouping as a working classification, and drew a tentative distinction between Cambalidae and Iulomorphidae. In both families the anterior gonopods are thought to deliver sperm, but the posterior gonopods are better developed in Cambalidae. Twenty years later, after examining the eastern Australian cambaloids he had collected in 1980, Jeekel retained the two-family classification and offered another diagnostic character:

“*The main character used for distinguishing the Cambalidae from the Iulomorphidae is the presence of a well developed flagellum in the anterior gonopods. It remains to be seen if this character is of sufficient importance to separate the two groups. In the family Julidae genera with and without a flagellum may be quite closely related.*” (Jeekel 2006: 65)

Korsós and Read (2012) decided that presence/absence of a flagellum was not of sufficient importance, and placed all Southern Hemisphere cambaloid genera in Iulomorphidae:

“...*we prefer here to consider all the following 15 genera in the family Iulomorphidae. The only diagnostic difference for separating Iulomorphidae from Cambalidae was the absence of a flagellum in the anterior gonopods (Jeekel 2006a) but this can be a homoplastic, convergent character state as has been shown for the family Julidae (Enghoff 1981, Read 1990). Given this situation, we think it is plausible to combine the very similar Southern Hemisphere (i.e. Gondwanan) elements into one family.*” (Korsós and Read 2012: 44)

Since “Gondwanan” and “Southern Hemisphere (-dwelling)” are not synapomorphies, this action left Iulomorphidae sensu Korsós & Read, 2012 not diagnosable on morphology. I accepted Iulomorphidae sensu Korsós & Read, 2012 for *Amastigogonus* Brölemann, 1913, *Atelomastix* Attems, 1911 and *Equestrigonus* Mesibov, 2017 (Mesibov 2017a). However, like Jeekel I have examined two distinct groups of Australian cambaloids: flagella-bearing taxa with relatively well-developed posterior gonopods and non-flagella-bearing taxa with greatly reduced posterior gonopods. The two groups differ ecologically as well, at least in southeast mainland Australia and Tasmania. The first group is most abundant in leaf litter and is common in dry forest and woodland, while the second is mainly associated with rotting wood in higher rainfall areas.

The higher classification of Australasian cambaloids may be resolved in future with the inference of a molecular phylogeny of the group. In the meantime, although I do not wish to further confuse the already muddled classification of the cambaloids, I would like to formalise the distinction between the two Australasian cambaloid groups. I am therefore restoring the Southern Hemisphere genera to the families in which they were placed by Hoffman (1980), with the addition of new Australian genera as assigned by Jeekel (2006, 2009) and Mesibov (2017a), and with species numbers from MilliBase (http://www.millibase.org/; accessed 2 January 2019) amended following Korsós and Read (2012) and Mesibov (2017a, 2017b) as follows:

**Southern Hemisphere genera of Cambalidae Bollman, 1893**:

*Apocoptogonus* Jeekel, 2006 (2 spp; eastern Australia)

*Dimerogonus* Attems, 1903 (1 Australian sp; eastern Australia)

*Eumastigogonus* Chamberlin, 1920 (11 spp; New Zealand)

*Euryischiogonus* Jeekel, 2009 (1 sp; eastern Australia)

*Proscelomerion* Verhoeff, 1924 (1 sp; eastern Australia)

*Stenischiogonus* Jeekel, 2009 (1 sp; eastern Australia)

*Zinagon* Chamberlin, 1957 (1 sp; southern Chile)

**Genera of Iulomorphidae Verhoeff, 1924**:

*Amastigogonus* Brölemann, 1913 (11 spp; Tasmania)

*Atelomastix* Attems, 1911 (30 spp; western and eastern Australia, including Tasmania)

*Dinocambala* Attems, 1911 (1 sp; western Australia)

*Equestrigonus* Mesibov, 2017 (1 sp; Tasmania)

*Iulomorpha* Porat, 1872 (ca 18 spp; southern Africa; excluding the three Australian “*Iulomorpha*” of Silvestri (1897), which may be cambalids)

*Merioproscelum* Verhoeff, 1924 (1 sp; eastern Australia)

*Podykipus* Attems, 1911 (3 spp; western Australia)

*Samichus* Attems, 1911 (2 spp; western Australia)

*Thaumaceratopus* Verhoeff, 1924 (2 spp; eastern Australia)

*Victoriocambala* Verhoeff, 1944 (2 spp; eastern Australia)

Cambalidae as recognised here and by Hoffman (1980, 1982) and Jeekel (2006, 2009) is a temporary, place-holding taxon and may not be a natural group. It can be distinguished from Iulomorphidae within the Australasian cambaloids by the much greater development of the posterior gonopods and by the presence of a long, very slender flagellum arising medially near the base of the anterior gonopod coxa.

In this paper I describe a new genus and three new species of Cambalidae from Tasmania. I also describe an interesting new species of Tasmanian Iulomorphidae and erect for it a new genus.

## Materials and methods

All specimens are preserved in 80% ethanol in their respective repositories.

Photomicrographs were taken with a Canon EOS 1000D digital SLR camera mounted on a Nikon SMZ800 binocular dissecting microscope equipped with a beam splitter. Measurements were made to the nearest 0.1 mm with the same microscope using an eyepiece grid and a reference scale. Photomicrographs used in the figures are focus-stacked composites prepared with Zerene Stacker 1.04. Scanning electron microscope images were acquired digitally using an Hitachi SU-70; specimens were examined after air-drying and sputter-coating with a minimal layer of platinum, then removed from stubs and returned to alcohol. The gonopods of the iulomorphid holotype were temporarily mounted in 1:1 glycerine:water and imaged using an eyepiece video camera mounted on an Amscope binocular microscope. Preliminary drawings of the gonopods were traced from printed copies of images, and drawings were then edited by reference to the actual specimens. Images and drawings were prepared for publication using GIMP 2.8.

Maps were drawn with QGIS 2.4. Latitude/longitude figures in the text (all based on the WGS84 datum) are given in decimal degrees to four decimal places, together with a spatial uncertainty. In some cases, collecting site locations have been upgraded from UTM grid references (on original labels, with the AGD66 datum), based on advice from collectors and the latest digital mapping of Tasmania. The spatial uncertainty figure covers the likely error in the location upgrade as well as my estimate of the likely error in the original location. All specimen records referred to in the text are in Supplement 1.

I follow Enghoff et al. (1993) in counting trunk rings by excluding the telson and giving podous + apodous ring counts, e.g. “(55+1) rings”, and I give the upper limits of the count ranges I observed rather than count frequencies.

Abbreviations:

**QVMAG** Queen Victoria Museum and Art Gallery, Launceston, Australia

**TMAG** Tasmanian Museum and Art Gallery, Hobart, Australia

**ZMUC** Zoological Museum, Natural History Museum of Denmark, Copenhagen, Denmark

## Results

### Order Spirostreptida Brandt, 1833

#### Family Cambalidae Bollman, 1893

##### Tasmanocambala gen. n.

Taxon classificationAnimaliaSpirostreptidaCambalidae

http://zoobank.org/667CC914-B9F5-4489-9DEB-747074BC19E1

###### Type species.

*Tasmanocambalagreeni* sp. n., by present designation.

###### Name.

“Tasmano”, combining form of Tasmania + *Cambala*, type genus of Cambalidae; feminine gender.

###### Diagnosis.

Differs from the other seven Southern Hemisphere cambalid genera by the anterior gonopod telopodite ending in a thin tab just anterior to an apical, transverse comb of setae; from *Dimerogonus* and *Eumastigogonus* in having a smoothly rounded apex of the coxal process, without a medial extension; from *Proscelomerion* in lacking a pseudoflagellum and in having a rounded rather than acuminate tip on the coxal process; from *Apocoptogonus* and *Euryischiogonus* by the flagellum not having a bifurcate tip; from *Stenischiogonus* by the lack of a distinct medial lobe on the tip of the anterior gonopod telopodite; and from *Zinagon* by the male leg 1 femur being much wider than the more distal three podomeres and by the anterior section of the posterior gonopod lacking a needle-like extension.

###### Description.

Living animals grey-black, in life with lighter-coloured annular band at rear of metazonite. Male/female midbody diameters to ca 2.5/2.9 mm; trunk ring counts to 55/56.

**Figure 1.**
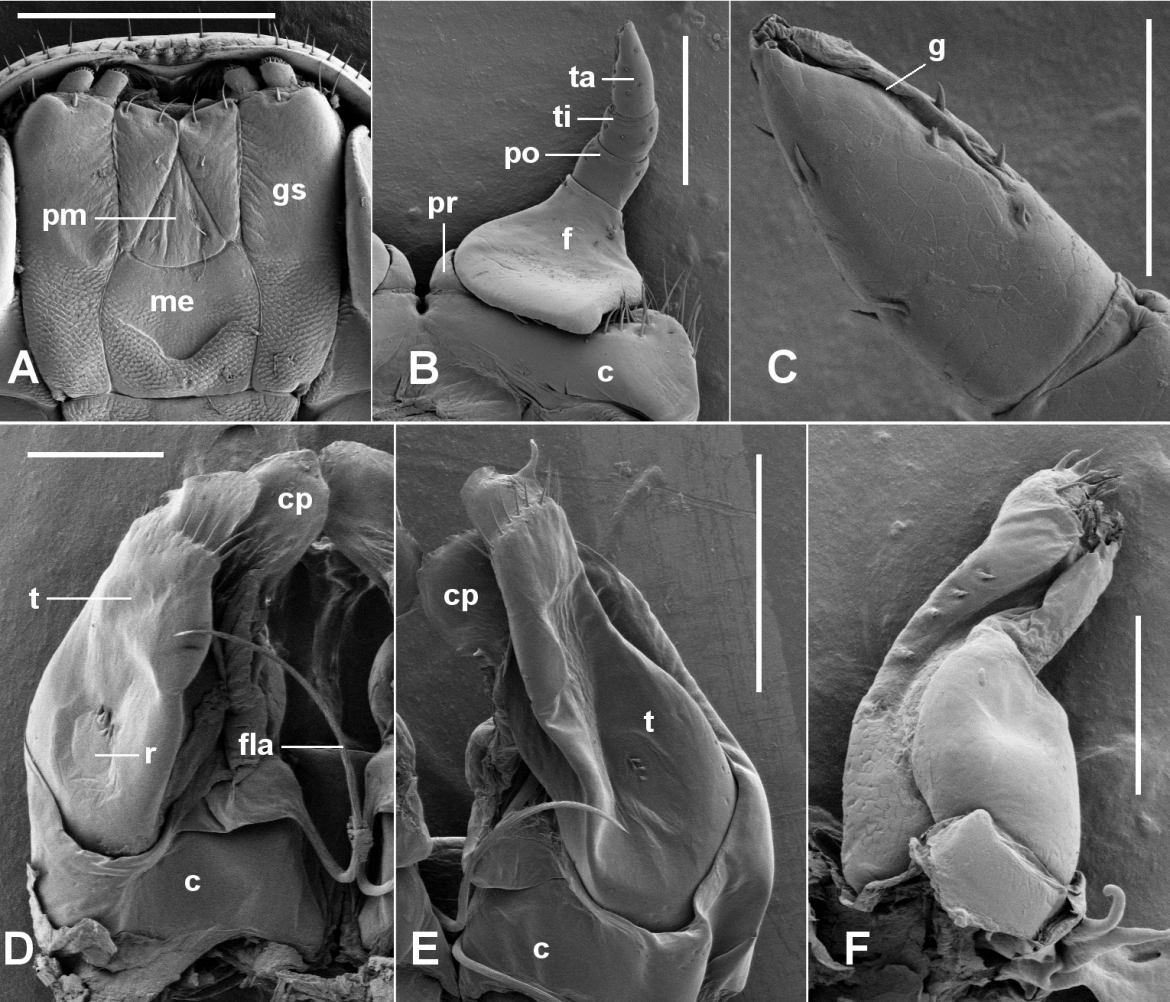
*Tasmanocambalagreeni* gen. n. et sp. n. (**A, B, D, F**) and *T.tasmanica* sp. n. (**C, E**). **A** gnathochilarium; QVM:2017:23:0028 **B** left leg 1, anterior view; QVM:2017:23:0034 **C** tarsus of right leg 1, anterior view; QVM:2017:23:0091 **D** right anterior gonopod, posterior view; QVM:2017:23:0028 **E** left anterior gonopod, posterior view; QVM:2018:23:0075 **F** left posterior gonopod, anterior view; QVM:2017:23:0028. **c** = coxa, **cp** = coxal process, **f** = femur, **fla** = flagellum, **g** = medial groove on tarsus, **gs** = gnathochilarial stipes, **me** = mentum, **pm** = promentum, **po** = postfemur, **pr** = prefemur, **r** = “rudimentary terminal podomere” of Korsós and Read (2012), **t**= telopodite, **ta**= tarsus, **ti** = tibia. Scale bars: 0.5 mm (**A**); 0.25 mm (**B, E**); 0.1 mm (**C, D, F**).

**Figure 2.**
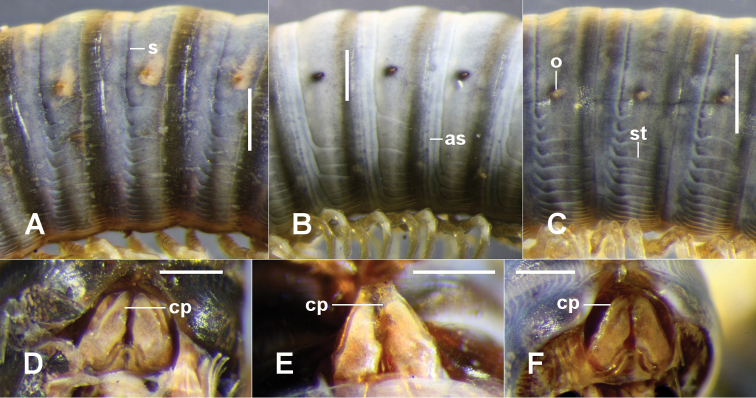
**A–C** Left lateral views of midbody rings of *Tasmanocambalagreeni* gen. n. et sp. n. (**A** holotype), *T.tasmanica* sp. n. (**B** paratype ex QVM:23:54461) and *T.taylori* sp. n. (**C** holotype). **D–F** Ventral views of gonopod complex in situ of *T.greeni* gen. n. et sp. n. (**D** QVM:2018:23:0080), *T.tasmanica* sp. n. (**E** paratype ex QVM:23:54461) and *T.taylori* sp. n. (**F** paratype ex QVM:2017:23:0057). **as** = annular stria, **cp** = coxal process, **o** = ozopore, **s** = suture, **st** = horizontal stria. Scale bars: 0.5 mm (**A–E**), 0.25 mm (**F**).

Head smooth apart from sparse setae on clypeus. Antenna reaching ring 2 dorsally when extended, relative antennomere lengths (3,6)>(2,4,5), 6^th^ antennomere widest, 4 apical cones. Ocelli of older individuals in 3 or 4 rows, posterior row longest with 7+ ocelli. Collum half-moon-shaped in dorsal view; corners broadly rounded. Gnathochilarium (Fig. 1A) with gnathochilarial stipetes well separated posteriorly by wide mentum; a broad medial depression in the mentum, deepest posteriorly, with anteriorly concave posterior margin; promentum triangular with base of triangle convex. Trunk rings (Fig. 2A-C) smooth, shiny; prozonite demarcated from metazonite by shallow constriction containing suture dorsally; suture turning posteriorly just ventral to ozopore, becoming dorsalmost of parallel series of horizontal striae on lateral and ventral portions of metazonite; limbus short, lamellar. Ozopores beginning on ring 6 at ca 1/2 ring height, slightly higher on subsequent rings; ozopores very small, round, in small, slight depressions at slightly less than 1/2 the distance between suture and posterior metazonite margin. Telson with dorsal margin of preanal ring only slightly produced, not forming distinct epiproct; hypoproct margin broadly paraboloid. Midbody legs ca 1/2 ring diameter in length; relative podomere lengths (prefemur=tarsus)>femur>postfemur>tibia; claw ca 1/2 as long as tarsus.

Male leg 1 (Fig. 1B) on undivided sternite; coxae fused with sternite but demarcation clearly visible; coxa mediolaterally widened and anteroposteriorly flattened, a few long setae in 1 or2 transverse rows on distolateral margin of coxa; prefemur very short, wide, subcylindrical; femur wide, tapering distally, extended basally on anterior surface as bluntly rounded process overlapping both prefemur and coxa; postfemur and tibia subcylindrical; tarsus subcylindrical and tapering distally, with deep, narrow groove medially (Fig. 1C); claw absent (or in some specimens small, malformed, on one leg of a pair); a few very small setae on distalmost 4 podomeres; relative widths prefemur > femur >> postfemur > tibia > tarsus; relative lengths (femur = tarsus) > postfemur > tibia > prefemur.

Aperture on ring 7 (Fig. 2D-F) cordate (apex to rear), the lateral margins slightly raised. Anterior and posterior gonopods forming small, compact structure, tilted posteriorly in ring 7. Coxa of anterior gonopod (Figs 1D, E; 2) about as long as telopodite or a little shorter, anteroposteriorly flattened and with large posterior concavity holding telopodite; apex rounded and very thin, directed distomedially; flagellum not bifurcate, arising medially on coxal base and curving first posterobasally, then distally, then anteriorly. Telopodite of anterior gonopod (Fig. 1D, E) not as wide as coxa, tapering distally, posterolaterally slightly excavate with a few very short setae in deepest portion of excavation near base (“rudimentary terminal podomere” of Korsós and Read (2012)); telopodite ending in translucent, rounded tab with comb of setae just posterior to tab, the setae shorter than tab. Posterior gonopods (Fig. 1F) reaching ca 2/3 height of anterior gonopods; anteriorly divided by deep, oblique groove into anterolateral and posteromedial sections, subequal in height; anterolateral section tapering at ca 1/2 section height from wide base to apically rounded lamina; posteromedial section with apex stout, bluntly rounded and tipped with sparse brush of short setae, and with row of very short setae along anteromedial margin of section.

Females like males in overall appearance but noticeably stouter; vulvae not examined.

###### Distribution.

So far known only from Tasmania, Australia.

###### Remarks.

Males of *Tasmanocambala* gen. n. species are identifiable by examination of the tip of the anterior gonopod, even with the gonopods lying in situ in ring 7: there is a terminal fringe of setae apparent behind a thin, translucent, anterior tab. The type species is likely to be a species complex (see Remarks on the type species) and the taxonomy of this genus would greatly benefit from genetic analysis.

The deep medial groove on the male leg 1 tarsus (Fig. 1C) is hard to detect with optical microscopy. It may be an autapomorphy for the genus, or it may be present in other cambaloid millipedes but overlooked by describers.

##### Tasmanocambala greeni sp. n.

Taxon classificationAnimaliaSpirostreptidaCambalidae

http://zoobank.org/AE0BA87A-060C-405A-818A-CD30E374AB9E

Figs 1A, B, D, F ; 2A, D

###### Holotype.

Male in pieces, gonopod complex in genitalia vial, Maggs Mountain Road, Tasmania, -41.6908, 146.2075 ±2 km, ca 450 m a.s.l., 8 October – 6 November 1979, R.H. Green, QVMAG QVM:2017:23:0006.

###### Paratypes.

In QVMAG: 1 female, same general locality and collector as holotype but compartment 2 turnoff, -41.7264, 146.1872 ±1 km, ca 880 m a.s.l., 17 May 1979, QVM:2017:23:0005; 2 females, same details but 4 February 1980, QVM:2017:23:0007; 1 male, 6 females, same details but from “tussock corner”, -41.6908, 146.2075 ±2 km, 18 March 1980, QVM:2017:23:0008; 2 males, same details but from “plateau rainforest”, 3 December 1980, QVM:2017:23:0009; 1 female, same details but from “site F”, -41.7269, 146.1878 ±1 km, ca 880 m a.s.l., 14 January 1981, QVM:2017:23:0010; 1 male, same details but 23 September 1981, QVM:2017:23:0277; 1 female, same details but 20 February 1989, QVM:2017:23:0020; 3 females, same details but from “site E”, -41.7258, 146.1867 ±1 km, ca 800 m a.s.l., 9 February 1982, QVM:2017:23:0011; 2 females, same details but 21 February 1984, QVM:2017:23:0012.

###### Other material.

89 males and 76 females from 44 sites other than the type locality, in QVMAG, TMAG and ZMUC; see Supplement 1 for details.

###### Description.

As for the genus, with the following details: male/female to 55+1/56+1 rings, 1.9/2.3 mm in midbody diameter. Anterior gonopod tip with 3–5 setae behind apical tab, on medial side (Fig. 1D).

###### Distribution.

Widespread in Tasmania but not yet recorded from the Midlands or the Northeast (Fig. 5A).

###### Name.

In honour of Robert “Bob” Green (1925–2013), Tasmanian zoologist and former Curator of Zoology at QVMAG. Green collected the type specimens of *T.greeni* sp. n. during his 15-year study of the impact of logging operations at Maggs Mountain in northwest Tasmania.

###### Remarks.

This species is likely to be a species complex, as there are geographically correlated variations in body size and in the length, position and distinctiveness of the horizontal striae on the trunk rings. However, I have not been able to observe any consistent, corresponding differences in gonopod structure. I chose the type specimens from the largest form in the putative species complex; this larger form mainly occurs in mid – to high-elevation areas in northwest and central Tasmania.

##### Tasmanocambala tasmanica sp. n.

Taxon classificationAnimaliaSpirostreptidaCambalidae

http://zoobank.org/F5885BDE-74BE-4A88-8CDF-B6DEE34D488B

Figs 1C, E ; 2B, E

###### Holotype.

Male, Mt Gnomon, Tasmania, -41.1777, 146.0289 ±25 m, 290 m a.s.l., 16 April 2016, R. Mesibov, QVMAG QVM:2018:23:0116.

###### Paratypes.

In QVMAG: 2 males, 4 females, details as for holotype, QVM:23:54461; 1 male, same locality and collector, -41.1739, 146.0344 ±100 m, 14 February 1996, QVM:2017:23:0057; 3 males, 3 females, same locality and collector, -41.1775, 146.0285 ±25 m, 300 m a.s.l., 5 February 2017, QVM:2017:23:0091.

###### Other material.

53 males, 47 females from 42 sites other than the type locality, in QVMAG; see Supplement 1 for details.

###### Name.

This species appears to be endemic to Tasmania.

###### Diagnosis.

Distinguished from *T.greeni* n. sp. and *T.taylori* n. sp. by the annular striae on the prozonite and by the larger number of setae in the apical comb on the anterior gonopod telopodite.

###### Description.

As for the genus, with the following details: in life, legs distinctly red-coloured in life (colour fades in alcohol) and strongly contrasting light-coloured annular band at rear of metazonite; male/female to 52+1/56+1 rings, 2.5/2.9 mm in midbody diameter. Trunk rings with variable number of annular striae on prozonite anterior to suture, most obvious ventrally (Fig. 2B). Anterior gonopod coxa (Figs 1E, 2E) with broad tip, usually wider than in *T.greeni* n. sp., usually shorter than telopodite; setal comb behind apical tab extending full width of telopodite tip and with 7-10 setae; tab sometimes with small finger-like projection laterally.

###### Distribution.

East of Tyler’s Line (Mesibov 1994) in the Northwest, but apparently absent from the northeast of the main island and from islands in Bass Strait (Fig. 5B). The polydesmidan *Tasmanodesmushardyi* Chamberlin, 1920 is similarly distributed (Mesibov 2004).

###### Remarks.

*Tasmanodesmustasmanica* n. sp. is the largest cambalid species in Tasmania and could be confused at first glance with the similar-sized iulomorphid *Equestrigonustasmaniensis* Mesibov, 2017. The two spirostreptidans have not yet been found to co-occur, but their ranges may overlap in wet forest south of Wynyard and near Blessington.

*Tasmanodesmustasmanica* sp. n. is very abundant in the Dial Range south of Penguin (i.e., around the type locality), where it can readily be found in and under damp leaf litter in wet eucalypt forest at any time of year.

##### Tasmanocambala taylori sp. n.

Taxon classificationAnimaliaSpirostreptidaCambalidae

http://zoobank.org/3E485A0D-02E3-4948-BF3B-ADD70407500E

Fig. 2C, F

###### Holotype.

Male in 3 pieces, anterior portions in genitalia vial, Badgers Hill, Flinders Island, **Tasmania**, -40.0275 148.0233 ±100 m, ca 200 m a.s.l., 31 August 1993, R.J. Taylor, QVMAG QVM:2018:23:0115.

###### Paratypes.

In QVMAG: 2 females, details as for holotype, QVM:2017:23:0041.

###### Other material.

18 males, 41 females and 3 juveniles from 11 other sites on Flinders Island and 4 sites on Prime Seal Island, in QVMAG and TMAG; see Supplement 1 for details.

###### Name.

In honour of Robert Taylor, collector of the type specimens. During his 13 years in Tasmania, Taylor instigated and managed a number of forest conservation projects that employed the author and other local zoologists as field workers and specimen processors. Material collected for those projects has been a valuable taxonomic resource for the author and others, and will continue to be valuable for years to come.

###### Diagnosis.

Distinguished from *T.tasmanica* n. sp. by the absence of annular prozonite striae and from *T.greeni* n. sp. by the strong mediad curvature and greater narrowing of the tip of the anterior gonopod coxa.

###### Description.

As for the genus, with the following details: male/female to 55+1/52+1 rings, 1.4/1.8 mm in midbody diameter. Anterior gonopod with tip of coxa curving strongly medially and narrowing (Fig. 2F); 3-4 apical telopodite setae behind tab on medial side.

###### Distribution.

So far known from Flinders and Prime Seal Islands at the eastern end of Bass Strait between Tasmania and Victoria (Fig. 5A).

###### Remarks.

*Tasmanodesmustaylori* sp. n. is not greatly different from central Tasmanian forms of *T.greeni* n. sp., and the narrowing of the tip of the anterior gonopod coxa is variable from specimen to specimen.

#### Suborder Epinannolenidea Chamberlin, 1922

##### Family Iulomorphidae Verhoeff, 1924

###### Talomius gen. n.

Taxon classificationAnimaliaSpirostreptidaIulomorphidae

http://zoobank.org/670C1ED1-7903-481B-B3E4-2249DEE9641C

####### Type species.

*Talomiusweldensis* sp. n., by present designation.

####### Name.

Anagram of “tasm” from “Tasmania” and “iulo” from “Iulomorphidae”; masculine gender.

####### Diagnosis.

Distinguished from all other genera of Iulomorphidae by the male first legs having a reduced prefemur, but with the four more distal podomeres appearing as in normal walking legs, including a normal claw on the tarsus; and distinguished from the other nine Australian iulomorphid genera by the small size of the gonopods relative to ring 7 diameter, by the strong medial curvature of the coxal process on the anterior gonopod, and by the bare, posteriorly curving apex of the anterior gonopod telopodite terminating in the opening of the prostatic groove.

####### Description and distribution.

As for the type species.

###### Talomius weldensis sp. n.

Taxon classificationAnimaliaSpirostreptidaIulomorphidae

http://zoobank.org/8137B3A4-3385-4A1A-9E83-7F07B8427957

Figs 3 , 4

####### Holotype.

Male, dissected, with pieces in genitalia vials (see Remarks), Mt Weld altitudinal transect, **Tasmania**, – 42.9981 146.6167 ±100 m (originally UTM 55G “468762 5239322”, GDA94 datum), ca 600 m a.s.l., pitfall 5U emptied 28 March 2012, M. Driessen and N. Doran, QVMAG QVM:2018:23:0118.

####### Paratypes.

In QVMAG: 2 males, dissected and without gonopods (see Remarks), details as for holotype, QVM:23:54522.

####### Other material.

None.

####### Name.

For the type locality, Mt Weld.

####### Description.

**Figure 3.**
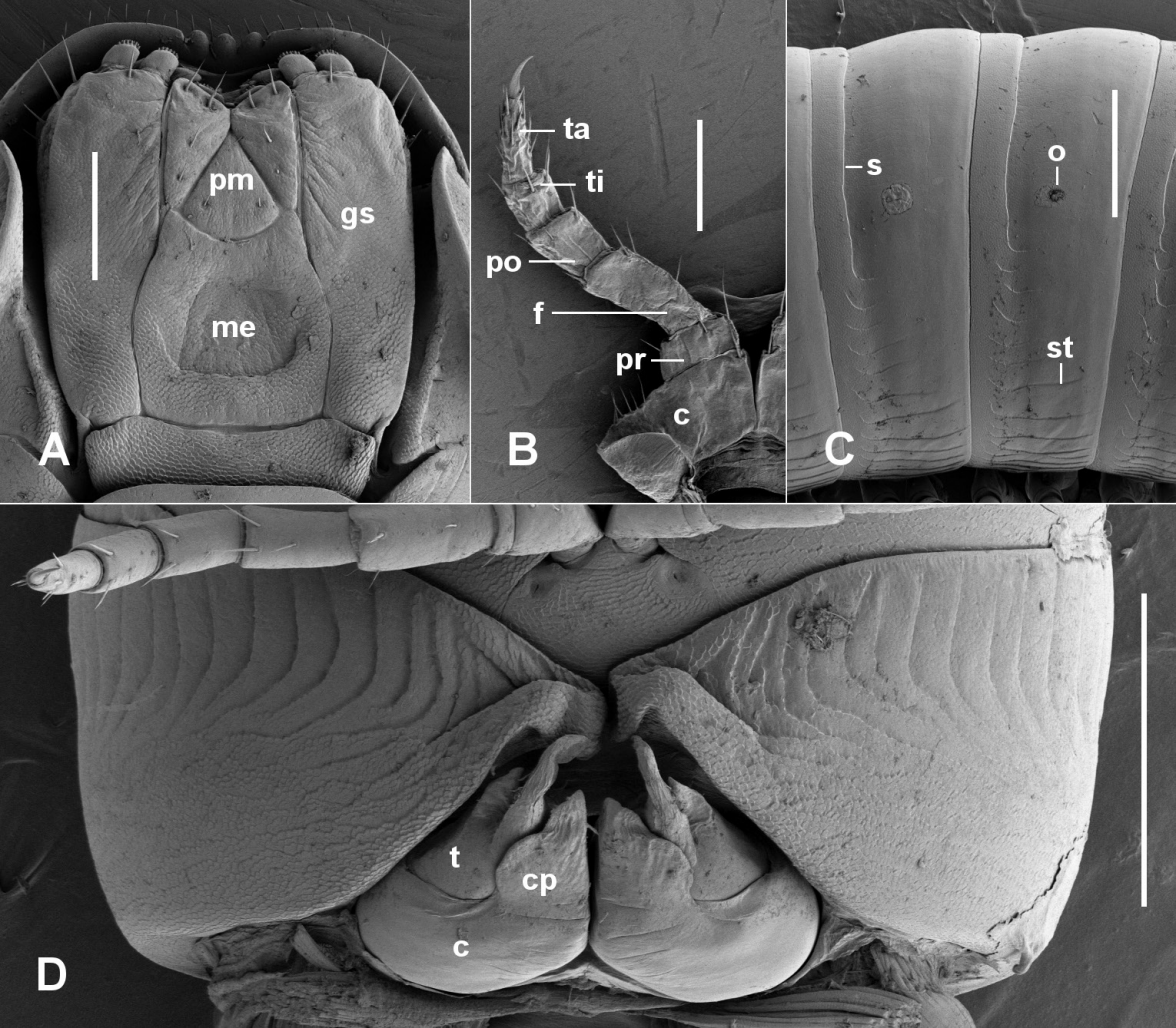
*Talomiusweldensis* gen. n. et sp. n. , holotype. **A** gnathochilarium **B** right leg 1, anterior view (leg shrivelled due to drying) **C** midbody ring, left lateral view **D** ring 7, ventral view. **c** = coxa, **cp** = coxal process, **gs** = gnathochilarial stipes, **me** = mentum, **pm** = promentum, **o** = ozopore, **s** = suture, **st** = horizontal stria, **t** = telopodite. Scale bars: 25 mm (**A**); 0.5 mm (**B–D**).

In alcohol, specimens grey-brown with lighter annular band at rear of metazonite. Largest male (paratype) with 36+4 body rings, 1.9 mm midbody diameter. Head smooth, clypeus moderately setose. Ocellar area lenticular; ca 20 ocelli in 4 rows in largest male (paratype), dorsal > ventral 6,6,5,3. Antennae short, just reaching rear of ring 2 when extended dorsally; relative antennomere lengths (2=3=6)>(4=5); antennomere 6 widest; 4 apical cones. Gnathochilarium (Fig. 3A) with lateral edges of mentum slightly convex; mentum wider than combined lingual plates, anterior edge strongly concave, posteriorly with wide medial depression, the posterior lip of the depression sharply defined, broadly “U”-shaped; promentum triangular with base of triangle convex. Collum strongly convex, almost symmetrical around transverse axis, the corners bluntly acuminate. Ring 2 with ventrolateral margin slightly produced, rings 3 and 4 similarly produced but less so. Prozonites and metazonites (Fig. 3C) smooth, shiny; shallow waist with weakly defined suture line, most distinct dorsally; indistinct, fine horizontal striae in lower 1/3 of trunk rings, anteriorly bending upwards and extending anteriorly onto the prozonite, past an imaginary continuation of the suture. Limbus lamellar, undivided. Ozopore on ring 6 at ca 1/2 ring height, slightly higher on subsequent rings; ozopores small, round, located ca 1/3 the distance between suture and posterior metazonite margin. Telson with preanal ring smooth; posterior margin only slightly extended over anal valves medially, not forming distinct epiproct; hypoproct with gently convex dorsal margin. Midbody legs ca 2/3 ring diameter in length; relative podomere lengths (prefemur=femur=tarsus)>postfemur>tibia.; claw ca 1/2 as long as tarsus. No prefemoral tab on any legs.

Leg 1 (Fig. 3B) with coxa laterally produced, anteroposteriorly flattened, with a few setae on distal margin lateral to prefemur; prefemur reduced, with normally long setae; distal podomeres as in walking legs, with normally long setae; relative podomere lengths femur>tarsus>postfemur>tibia>>prefemur; claw ca 1/2 tarsus length. Leg 2 with penis forming a small plate at posterodistal end of elongated coxa.

**Figure 4.**
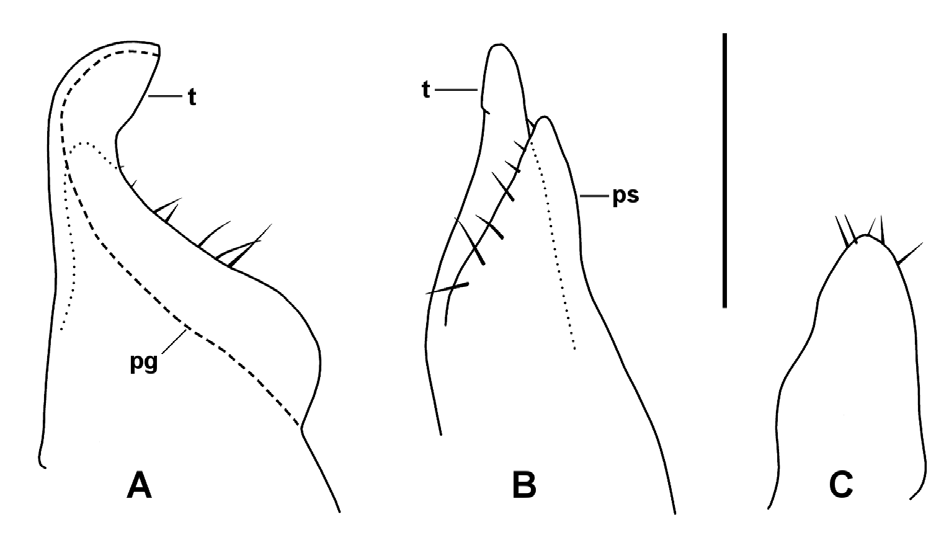
*Talomiusweldensis* gen. n. et sp. n., holotype. Left anterior gonopod, medial (**A**) and posterior (**B**) views, and right posterior gonopod (**C**), posterior view. Dashed line (**pg**) indicates course of prostatic groove, dotted lines indicate outline of hidden structure. **pg** = prostatic groove, **ps** = pseudoflagellum, **t** = telopodite. Scale bar: 0.25 mm (approximate).

Aperture (Fig. 3D) V-shaped (apex to rear), the margin thickened and slightly raised posteriorly. Gonopods (Figs 3D, 4) in situ forming small, compact complex strongly tilted posteriorly. Anterior gonopod coxa short, bulbous, cradling base of telopodite laterally; coxal process arising distomedially and extending as flattened tab curving posteriorly and partly sheltering telopodite medially. Telopodite (Fig. 4A, B) erect, taller than coxal process, broad basally and strongly tapered. the tip curving posteriorly; pseudoflagellum wide, branching off medially at ca 2/3 telopodite height, paralleling telopodite but not as high, broadly rounded at apex; posterior surface of telopodite with narrow, flattened ridge bearing a few setae, continuing distally on pseudoflagellum; prostatic groove running along posteromedial surface of telopodite base, curving anterodistally and following outer margin of telopodite, terminating at posteriorly directed telopodite tip.

Posterior gonopods (Fig. 4C) separate, less than 1/2 anterior gonopod height; cradled within coxal recess and partly sheltered distally by telopodite base; fingertip-shaped with flattened anteromedial surface distally and with 5 or 6 short apical setae.

####### Distribution.

**Figure 5.**
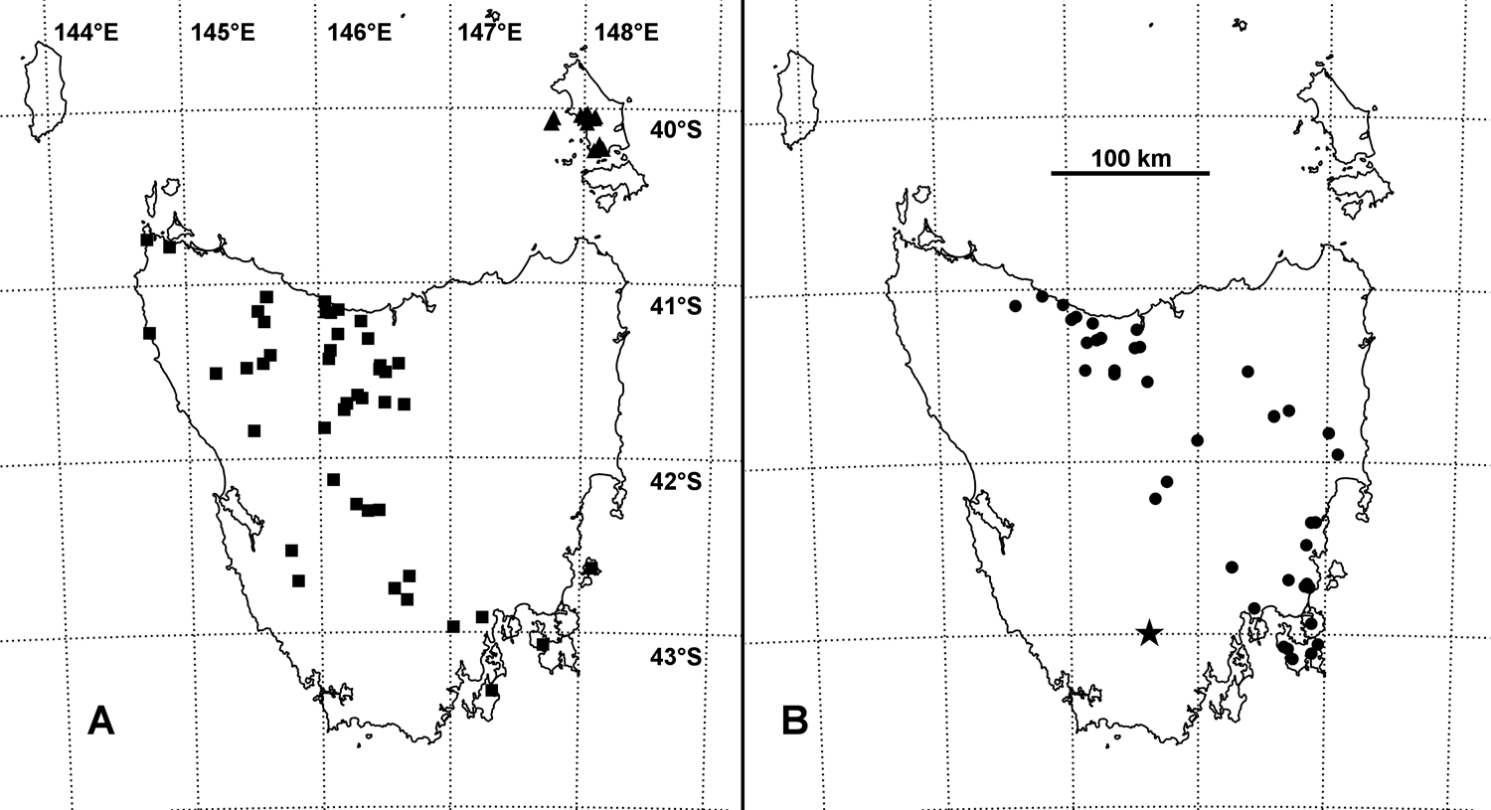
Known localities as of 31 December 2018 for *Tasmanocambalagreeni* gen. n. et sp. n. (**A** squares), *T.tasmanica* sp. n. (**B** circles), *T.taylori* sp. n. (**A** triangles) and *Talomiusweldensis* gen. n. et sp. n. (**B** star). Mercator projection; distance scale approximate.

Known only from the type locality (Fig. 5B).

####### Remarks.

When sorting spirostreptidan millipedes for an article on Tasmanian Iulomorphidae (Mesibov 2017a), I set aside the three Mt Weld males as “Cambalidae”, because the males had a small, compact gonopod complex like the Tasmanian cambalids described above, and the legs lacked the prefemoral tabs found in Australian Iulomorphidae. The males also had apparently ambulatory first legs, which so far as I am aware have not been reported before in any iulomorphids. When preparing the current article, I removed the gonopods of two of these “cambalids” and cleared and imaged one of the undissected complexes. Unfortunately, I then lost the two gonopod complexes, leaving only one of the three males intact. Rings 7 and 8 of that male were removed for SEM imaging of the gonopod complex (Fig. 3D), but with only a very thin coat of metal applied. The rings were returned to alcohol and the gonopods dissected and illustrated here; this specimen has been designated the holotype.

I regret not having additional material of *T.weldensis* n. sp. for study and description, but the type locality is in Tasmania’s southern mountain district, which in 2019 remains a remote and little-sampled wilderness area. The three known specimens of *T.weldensis* n. sp. were in pitfall traps emptied on 28 March 2012 at 600 m on Mt Weld, during a biological monitoring study along an altitudinal transect. They were among ca 50 *Amastigogonusverreauxii* (Gervais, 1847) (Iulomorphidae) in pitfalls emptied on the same day at the same elevation (*A.verreauxii* records in Mesibov (2006-2019)). The Mt Weld study generated its invertebrate samples in 2001-2002 and again in 2011-2012. I did not observe any other *T.weldensis* n. sp. specimens among the millipedes pitfall-trapped in the two sampling periods.

## Discussion

There are far fewer specimens of Cambalidae in Tasmanian museum collections than of Iulomorphidae. My field experience over 45 years in Tasmania is that this difference is not due to sampling bias, but reflects the patchy distribution of Cambalidae. Where they occur, however, Cambalidae are often abundant. This “abundant but patchy” distribution is also characteristic of procyliosomatid Sphaerotheriida in Tasmania. Hundreds of pill millipedes can be found in small patches of richly organic forest soil in some areas, while none are seen in apparently identical macro – and microhabitats nearby.

The patchiness of cambalids in Tasmania accords with what Jeekel (2006) observed on the Australian mainland: “During a collecting trip through New South Wales by the author and his wife between 27.X. and 12.XI.1980 only five sites out of 32 yielded representatives of the family. Since each sample consisted of a different species, it is obvious that Cambalidae are quite local in their occurrence. Possibly also their appearance in the upper layers of the soil is restricted to periods of favourable weather conditions. Under such conditions populations may be quite numerous” (Jeekel 2006, p. 65).

Jeekel found no Cambalidae in Victoria or Tasmania (Jeekel 2009, p. 83), but I have observed specimens of Victorian Cambalidae in Museums Victoria and have also collected cambalids in South Australia (specimens deposited in the South Australian Museum).

The almost complete lack of cambalid records in northeast Tasmania is curious, as the region has been intensively sampled for millipedes over many years and hundreds of iulomorphid Spirostreptida have been collected there (see fig. 7 in Mesibov (2017a)). Two, possibly introduced, cambalid species are known from Cuckoo Plantation, which is a long-established *Pinus* and *Eucalyptus* plantation near Scottsdale in northeast Tasmania (QVMAG specimen lots QVM:2017:23:0054 and QVM:2018:23:0003). These two species, one of which appears to be a *Dimerogonus*, may have been carried to Tasmania from mainland Australia on forestry equipment. I have not found additional specimens of either of these species in the Plantation or elsewhere in northeast Tasmania, despite careful recent searching. A third QVMAG specimen lot (QVM:2017:23:0016) contains a female and a juvenile of a possible cambalid from a logged forest area northeast of Goulds Country in the Northeast.

*Talomius* n. gen. is the fourth iulomorphid genus to be recognised in Tasmania. Like the two Tasmanian *Atelomastix* species, *T.weldensis* n. sp. may have a restricted distribution, but it also possible that the three spirostreptidans will be found to have substantial ranges when Tasmania’s southern and southwestern wilderness areas are carefully sampled for millipedes in future.

## Supplementary Material

XML Treatment for Tasmanocambala

XML Treatment for Tasmanocambala greeni

XML Treatment for Tasmanocambala tasmanica

XML Treatment for Tasmanocambala taylori

XML Treatment for Talomius

XML Treatment for Talomius weldensis

## Supplementary materials

Supplementary material 1 Specimen data for Tasmanian Spirostreptida Authors: Robert Mesibov Data type: occurrence Explanation note: Data file *Specimen_data_Mesibov_Tasmanian_Spirostreptida_2019.tsv* for 123 museum specimen lots of *Tasmanocambalagreeni* n. gen. n. sp., *T.tasmanica* n. sp., *T.taylori* n. sp. and *Talomiusweldensis* n. gen. n. sp. The file is a tab-separated table in UTF-8 encoding with the following 27 Darwin Core fields: institutionCode, catalogNumber, phylum, class, order, family, genus, specificEpithet, scientificName, typeStatus, organismRemarks, locality, country, stateProvince, decimalLatitude, decimalLongitude, geodeticDatum, coordinateUncertaintyInMeters, georeferenceSources, georeferencedBy, georeferenceRemarks, verbatimCoordinates, verbatimSRS, minimumElevationInMeters, recordedBy, eventRemarks and eventDate This dataset is made available under the Open Database License (http://opendatacommons.org/licenses/odbl/1.0/). The Open Database License (ODbL) is a license agreement intended to allow users to freely share, modify, and use this Dataset while maintaining this same freedom for others, provided that the original source and author(s) are credited.
